# Salsolinol Facilitates Glutamatergic Transmission to Dopamine Neurons in the Posterior Ventral Tegmental Area of Rats

**DOI:** 10.1371/journal.pone.0036716

**Published:** 2012-05-10

**Authors:** Guiqin Xie, Jiang-Hong Ye

**Affiliations:** Department of Anesthesiology, Pharmacology and Physiology, University of Medicine and Dentistry of New Jersey, New Jersey Medical School, Newark, New Jersey, United States of America; National Cheng Kung University, Taiwan

## Abstract

Although in vivo evidence indicates that salsolinol, the condensation product of acetaldehyde and dopamine, has properties that may contribute to alcohol abuse, the underlying mechanisms have not been fully elucidated. We have reported previously that salsolinol stimulates dopamine neurons in the posterior ventral tegmental area (p-VTA) partly by reducing inhibitory GABAergic transmission, and that ethanol increases glutamatergic transmission to VTA-dopamine neurons via the activation of dopamine D_1_ receptors (D_1_Rs). In this study, we tested the hypothesis that salsolinol stimulates dopamine neurons involving activation of D_1_Rs. By using whole-cell recordings on p-VTA-dopamine neurons in acute brain slices of rats, we found that salsolinol-induced increase in spike frequency of dopamine neurons was substantially attenuated by DL-2-amino-5-phosphono-valeric acid and 6, 7-dinitroquinoxaline-2, 3-dione, the antagonists of glutamatergic *N*-Methyl-D-aspartic acid and α-amino-3-hydroxy-5-methyl-4-isoxazolepropionic acid receptors. Moreover, salsolinol increased the amplitude of evoked excitatory postsynaptic currents (EPSCs) and the frequency but not the amplitude of spontaneous EPSCs. Additionally, SKF83566, a D_1_R antagonist attenuated the salsolinol-induced facilitation of EPSCs and of spontaneous firing of dopamine neurons. Our data reveal that salsolinol enhances glutamatergic transmission onto dopamine neurons via activation of D_1_Rs at the glutamatergic afferents in dopamine neurons, which contributes to salsolinol's stimulating effect on p-VTA dopamine neurons. This appears to be a novel mechanism which contributes toward rewarding properties of salsolinol.

## Introduction

The racemic mixture of salsolinol ((R) + (s)-salsolinol) is formed by nonenzymatic Pictet-Spengler condensation of dopamine with acetaldehyde, the major metabolite of ethanol in the brains of mammals [1], [2]. Salsolinol has been proposed to play a role in the etiology of alcoholism [3], and to the rewarding properties of ethanol [4]. Early animal studies revealed that salsolinol promotes alcohol drinking [5], [6]. More recent studies showed that rats self-administer salsolinol into posterior VTA (p-VTA), a key region in the brain reward system [7], [8], and that microinjection of salsolinol into the p-VTA of rats induces conditioned place preference [9]. Furthermore, such behaviors seem to depend on activation of dopaminergic (DA) neurons [8] and associated with enhanced dopamine levels in the ipsilateral nucleus accumbens shell [9]. With patch clamp techniques, we recently showed that salsolinol (0.01–1 µM) dose-dependently stimulates DA neurons in the p-VTA in acute midbrain slices of rats. Salsolinol decreased GABAergic synaptic transmission onto p-VTA DA neurons, and gabazine, an antagonist of GABA_A_ receptors substantially attenuated salsolinol-induced increase in firing rate of DA neurons. This phenomenon suggests that salsolinol stimulates DA neurons through a mechanism of disinhibition [10]. Interestingly, gabazine (10 µM) failed to completely abolish salsolinol-induced increase in DA neuron firing, indicating that other mechanisms may also be involved.

In addition to potent inhibitory GABAergic afferents [11], VTA DA neurons receive glutamatergic (Gluergic) inputs from diverse brain nuclei [12], [13] and from the Gluergic neurons in the VTA [14]. These diverse sources of Gluergic afferents may enable DA neurons to respond to a wide range of environmental stimuli. Such excitatory Gluergic synaptic input is a key component in the regulation of DA cell excitability [15], [16] and is known to play an important role in the actions of many drugs of abuse [17], [18] including ethanol [19]. Previous in vivo evidence indicates that activation of dopamine D_1_ receptors (D_1_Rs) can increase the release of glutamate in the VTA [20]. In recent in vitro studies, we have demonstrated that acute ethanol facilitates Gluergic transmission to VTA DA neurons via the activation of D_1_Rs at Gluergic afferents [21], [22]. This study was set to test the hypothesis that salsolinol stimulates DA neurons involving activation of D_1_Rs at Gluergic afferents.

## Methods

### Experimental procedures

All experiments were performed in accordance with the guidelines of the National Institutes of Health Guide for the Care and Use of Laboratory Animals and were approved by the Institutional Animal Care and Use Committee of the University of Medicine and Dentistry of New Jersey. All efforts were made to minimize animal suffering and to reduce the number of animal used. The experiments were performed on Sprague-Dawley rats aged 15–25 (20±1) postnatal (P) days.

### Slice preparation

The midbrain slices were prepared as described previously [23], [24]. Animals were anesthetized and then killed by decapitation. The brain was removed and a midbrain block (containing the VTA) was isolated. It was glued to the cutting stage of a VF-200 slicer (Precisionary Instruments Inc., Greenville, NC, USA). While the brain was kept in ice-cold glycerol-based artificial cerebrospinal fluid (GACSF) – containing 252 mM glycerol, 1.6 mM KCl, 1.2 mM NaH_2_PO_4_, 1.2 mM MgCl_2_, 2.4 mM CaCl_2_, 18 mM NaHCO_3_, and 11 mM glucose, and oxygenated with 95% O_2_/5% CO_2_. 200–250 µm thick slices were cut in the coronal plane. The slices (two per animal) were allowed to recover for at least 1 h in a holding chamber in regular artificial cerebrospinal fluid (ACSF), which has the same composition as GACSF, except that glycerol was replaced with 126 mM NaCl.

### Electrophysiological recording in midbrain slices

Cells in midbrain slices were visualized with an upright microscope (E600FN, Nikon, Tokyo, Japan) and near-infrared illumination. Electrical signals were obtained in whole-cell patch clamp technique with MultiClamp 700 A amplifiers (Molecular Devices Co., Union City, CA, USA), a Digidata 1320 A A/D converter (Molecular Devices Co.) and pCLAMP 9.2 software (Molecular Devices Co.). Data were filtered at 2 kHz and sampled at 5 kHz. The patch electrodes had a resistance of 4–6 MΩ when filled with the pipette solution containing (in mM) 125 K gluconate, 2.8 NaCl, 2 MgCl_2_, 0.6 EGTA, 10 HEPES, 2 ATP-Na, and 0.3 GTP-Na for whole-cell current clamp recordings. For whole-cell voltage clamp recordings the patch electrodes were filled with a pipette solution containing (in mM): 135 K gluconate, 5 KCl, 2 MgCl2, 10 HEPES, 2 Mg ATP, 0.2 GTP, and 2 QX-314. The pH was adjusted to 7.2 with Tris base and osmolarity to 300 mOsmol/L with sucrose. A single slice was transferred into a 0.4 ml recording chamber, where it was held down by a platinum ring. Warm carbogenated ACSF flowed through the bath (1.5–2.0 ml/min). All recordings were carried out at 32°C, maintained by an automatic temperature controller (Warner Instruments, Hamden, CT). For analyzing the changes in firing rate, we adapted the method used by Brodie's group [25]. Specifically, firing rate was calculated over 1-min intervals before administration of drugs and during the drug effect; peak drug-induced changes in firing rate were expressed as the percentage change from the control firing rate according to the formula [(FRD- FRC)/FRC] X 100, where FRD is the firing rate during the peak drug effect and FRC is the control firing rate. Thus, the change in firing rate is expressed as a percentage of the initial firing rate, which controls for small changes in firing rate, which may occur over time.

All EPSCs were recorded at a holding potential (Vh) of −70 mV. Physiological identification of DA neurons was based on the presence of hyperpolarizing current (I_h_), which was greater than 60 pA during a step from −50 to −100 mV, and the rate of spontaneous action potential activity (1–5 Hz) with spike widths ≥1.2 ms [21], [26], [27]. To evoke monosynaptic excitatory postsynaptic currents (eEPSCs), a bipolar stainless steel-stimulating electrode was placed 50–200 µm away from the recorded VTA neuron. Electrical stimuli (100–200 µs in duration) were applied at the rate of 0.05–0.1 Hz. Near the start of the recording, an input/output curve was obtained, and the stimulation was then set to 20–30% of the maximum, an intensity that evoked stable responses with no failures. Paired eEPSCs were elicited with a pair of identical stimuli separated by an interval of 50 ms. Series resistance was checked before and after the experiments by series resistance compensation. To measure input resistance, we applied a 5 mV, 400 ms hyperpolarizing pulse and divided 5 mV by the final value of the evoked current. The data would be discarded if series resistance (15–30 MΩ) or input resistance (300–500 MΩ) changed by more than 20% during the whole-cell recording.

### Data analysis

Spontaneous discharges and excitatory postsynaptic currents (EPSCs) were counted and analyzed with Clampfit 9.2 (Molecular Devices Co). Spontaneous or miniature EPSCs (sEPSCs and mEPSCs) were screened automatically (8 pA amplitude threshold), checked visually, and accepted or rejected according to their rise (<1.5 ms) and decay (<3.5 ms) times. The height of evoked eEPSCs (eEPSCs) was measured with Clampfit 9.2 and was used to calculate the paired pulse ratio (PPR  =  EPSC_2_/EPSC_1_), where EPSC_1_ and EPSC_2_ were evoked by the first and second stimuli at an interval of 50 ms. The eEPSC amplitude, PPR, and the frequency of sEPSCs and mEPSCs during and after drug applications were normalized to their mean value during the initial control period (>3 min). These data were used to depict summarized time courses (10–30 s per/bin). The baseline mean values were obtained during the initial control period and the mean values were obtained during drug application over a 2–3 min period at the peak of a drug response. Drug effects were expressed as % change (mean ± SEM) from a pre-drug control baseline. Paired or unpaired two-tailed t-test evaluated the statistical significance of various drug effects (% change from control baseline). Cumulative probability plots of the incidence of various inter-event intervals and amplitudes (for 50–1000 sEPSCs and mEPSCs), were recorded in control conditions and during drug applications to the same neuron, were analyzed with the Kolmogorov-Smirnov (K–S) test. In the figures, single eEPSCs or paired eEPSCs are averages of >10 successive traces. Values of p<0.05 were considered significant.

### Chemicals and Applications

1-Methyl-6,7-dihydroxy-1,2,3,4-tetrahydroisoquinoline hydrobromide (Salsolinol), bicuculline, DL-2-amino-5-phosphono-valeric acid (DL-APV), 6, 7-dinitroquinoxaline-2, 3-dione (DNQX), QX-314, tetrodotoxin (TTX) were obtained from Sigma-Aldrich Chemical Company (St Louis, MO, USA). (±)-SKF 83566 was from TOCRIS Bioscience (Ellisville, MO, USA). The 1000× stock solutions were aliquoted and then stored at −20, 4°C, or room temperature according to the catalog recommendations. APV (50 µM) and DNQX (20 µM) were dissolved in ∼0.1 N NaOH and other agents in deionized water. Drugs at final concentration were added to the superfusate.

## Results

### Salsolinol-induced increase in the firing rate of p-VTA DA neurons is substantially attenuated by antagonists of Gluergic NMDA and AMPA receptors

**Figure 1:**
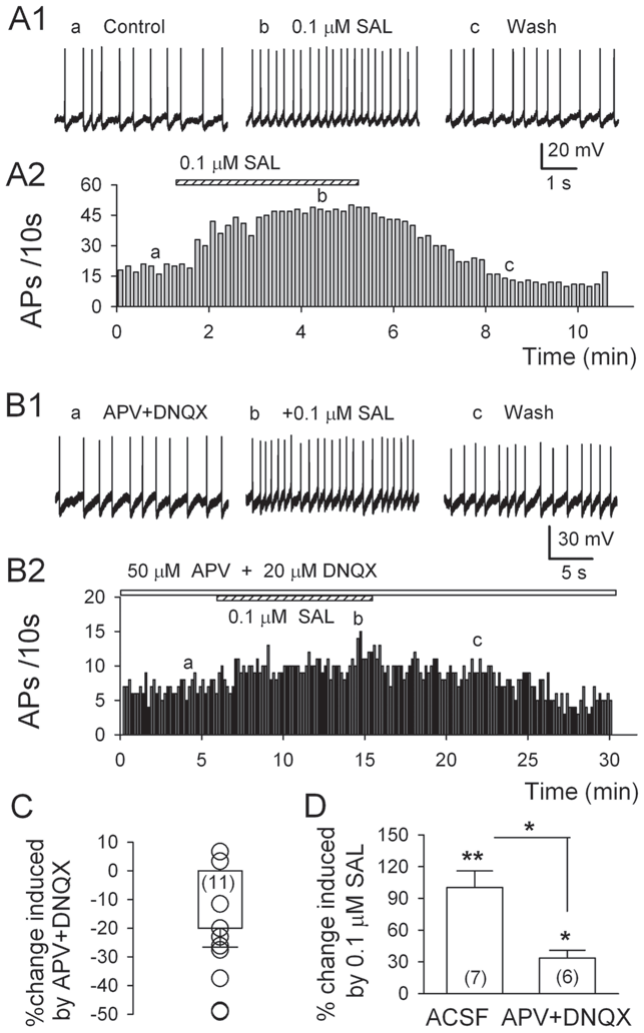
Salsolinol-induced stimulation of dopaminergic (DA) neurons is attenuated by APV and DNQX. A1, Traces illustrate spike discharge at the times indicated in A2. A2, Time course of the increase in the ongoing pacemaking firing rate, recorded from a current-clamped DA neuron in the posterior ventral tegmental area (p-VTA) of a rat, by 0.1 µM salsolinol. B1, Traces obtained at the times indicated in B2. B2, Time course of the effect of salsolinol on the firing rate of a DA neuron in the presence of APV (50 µM) and DNQX (20 µM), the antagonists of NMDA and AMPA receptors. C, Summary plot of decease in firing rate by APV + DNQX. D, Summary plot (means ± S.E.M.) of increase in firing rate of p-VTA DA neurons induced by salsolinol (0.1 µM) in ACSF is larger than that in the APV+DNQX. Numbers in bars indicate numbers of neurons tested. *P<0.05, **P<0.01, paired t-test for salsolinol vs. pre-salsolinol control. Unpaired t-test for salsolinol vs. APV+DNQX+salsolinol.

Whole-cell recording was performed in p-VTA-DA neurons in coronal midbrain slices of rats. We chose p-VTA because a previous in vivo study found that salsolinol has an effect on the posterior but not anterior VTA [8]. DA neurons were identified by their physiological and pharmacological properties as described by our many published documents [21], [23], [28]. Most DA neurons fired spontaneously in a regular pacemaker mode. As the basal firing rate varied, each neuron served as its own control. We recorded spontaneous firing for 5–10 min in the absence of salsolinol and then switched the perfusion to a solution containing salsolinol for 5–10 min before returning to control solution. Recently, we have shown that salsolinol (0.01–1 µM) dose-dependently increases the firing of DA neurons with a peak effect at 0.1 µM [10]. In keeping with this, salsolinol (0.1 µM) robustly increased the ongoing discharges recorded from current clamped DA neurons (by 100.3±15.6%, from 0.88±0.17 to 1.87±0.50 Hz, n = 7, P<0.001, by paired t test; Fig. 1A). This effect was reversible upon washout of salsolinol. An identical effect of salsolinol was observed using the loose-patch cell-attached configuration (data not shown).

Excitatory synaptic input mediated by glutamate is a key component in the regulation of DA cell excitability [15]. To assess whether salsolinol-induced excitation of DA neurons involves activation of Gluergic ionotropic receptors, we compared salsolinol's effects in the absence and presence of the mixture of APV (50 µM) and DNQX (20 µM), the antagonists of NMDA and AMPA receptors. APV+DNQX slightly but significantly reduced the firing rate of DA neurons to 80.0±6.4% of control (from 1.9±0.4 to 1.6±0.5 Hz, n = 11, P = 0.011; Fig. 1C). This indicates a significant Gluergic influence which tonically facilitates DA cells' activity. After the response to APV+DNQX stabilized, the addition of salsolinol (0.1 µM) increased the firing by 33.5±7.4 % (n = 6) (Fig. 1B). This increase was significant (P<0.05. vs. in the absence of salsolinol), but was substantially smaller than 100.3±15.6% that induced by salsolinol (0.1 µM) alone (Fig. 1D, P<0.05, student's unpaired t test). These data indicate that salsolinol-induced stimulation of DA neurons is mediated, at least partly, by Gluergic activity.

### Salsolinol increases the frequency but not the amplitude of spontaneous EPSCs (sEPSCs) in putative DA Neurons

**Figure 2:**
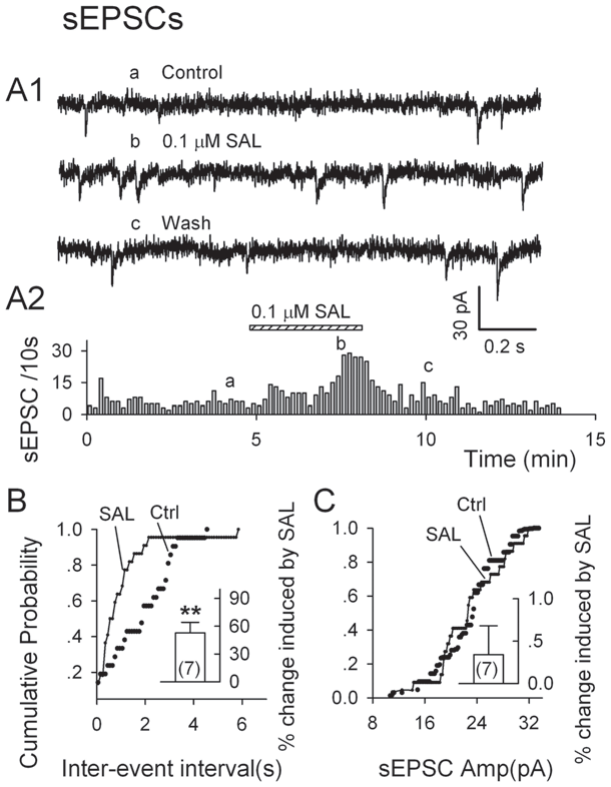
Salsolinol increases frequency of spontaneous EPSCs (sEPSCs) recorded from p-VTA DA neurons. EPSCs were recorded in the presence of bicuculline (10 µM) at a holding potential of −70 mV. A1, Traces obtained at the times indicated in A2. A2, Time course of 0.1 µM salsolinol-induced enhancement of sEPSC frequency in one experiment. B, C, Cumulative probability plots of data from the same cell show effects of salsolinol (0.1 µM) on sEPSC frequency (B) and amplitudes (C). Insets: mean data (± S.E.M., from seven neurons). *P<0.05, **P<0.01, paired t-test for salsolinol vs. pre-salsolinol control.

We then examined if salsolinol would affect Gluergic transmission onto DA neurons. We recorded sEPSCs in the presence of 10 µM bicuculline at a holding potential (Vh) of −70 mV. Their suppression by 20 µM DNQX (not shown) indicated that they were indeed Gluergic events mediated by AMPA receptors. Salsolinol (0.1 µM) increased their frequency by 50.9±12.4% (from 3.7±1.3 to 5.6±1.8 per 10 s, n = 7, P = 0.006, by paired t-test, Fig. 2A). This is further shown in Fig. 2B by the sharp increase in the probability of shorter intervals between successive sEPSCs (Kolmogorov-Smirnov, K–S test, P<0.01). After washout of salsolinol, sEPSC frequency returned to control levels (Fig. 2A). Conversely, salsolinol (0.1 µM) did not alter sEPSC amplitudes (control, 23.6±2.3 pA; salsolinol, 24.8±0.7 pA, n = 7, p = 0.53, paired t-test). The cumulative probability plot of amplitudes between successive sEPSCs also confirms this finding (K–S test, P>0.5, Fig. 2C). It is generally believed that a significant effect on sEPSC frequency without a concomitant effect on their amplitude is good evidence for an interference with the release mechanisms.

### Tetrodotoxin (TTX) abolishes Salsolinol's increases of spontaneous EPSCs in DA Neurons

**Figure 3:**
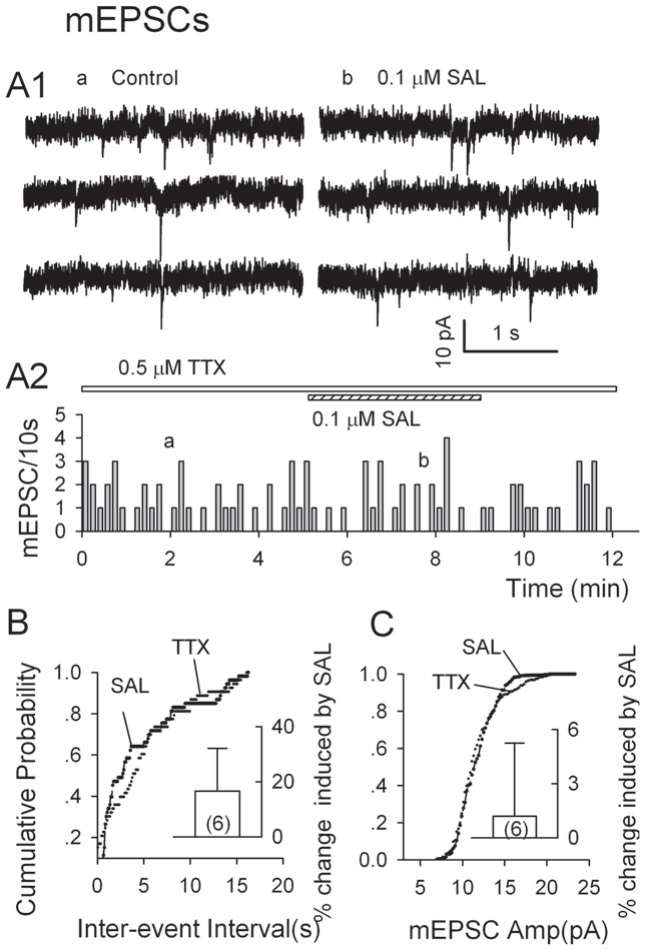
Tetrodotoxin (TTX) abolishes Salsolinol's increase of sEPSCs. The miniature EPSCs (mEPSCs) were recorded in p-VTA DA neurons at a holding potential of −70 mV, in the presence of bicuculline (10 µM) and TTX (0.5 µM). A1, Traces obtained at the times indicated in A2. (A2) Time course of salsolinol-induced changes of mEPSC frequency in one experiment. B, C, Cumulative probability plots of inter-event intervals between mEPSCs (B) and their amplitudes (C). Insets: mean data (± S.E.M., from six neurons) confirm the absence of significant effect of salsolinol (0.1 µM) on the frequency and the amplitude of mEPSCs. *P<0.05, paired t-test for salsolinol vs. pre-salsolinol control.

To further characterize the effects of salsolinol on the spontaneous EPSCs, we recorded miniature EPSCs (mEPSCs) in the presence of bicuculline (10 µM) and TTX (0.5 µM), which blocks action potential-generated events. TTX (0.5 µM) lowered the frequency of spontaneous EPSCs to 33.4±6.0% of control (from 2.7±0.7 to 0.8±0.4 per 10 s, n = 3, P = 0.008, paired t-test) without altering their amplitude (97.0±1.3% of control, from 15.3±3.1 to 14.9±3.1 pA, n = 3, p = 0.14, paired t-test). In the presence of TTX (0.5 µM), salsolinol (0.1 µM) did not significantly altered either the mEPSC frequency (by 16.7±15.5%, from 0.8±0.1 to 1.0±0.2 per 10 s, n = 6, P = 0.33, paired t-test), or their amplitude (by 1.2±4.0%, control, 20.7±1.3 pA; salsolinol, 20.9±1.5 pA, n = 6, P = 0.9, paired t-test; Fig.3A–C). Thus, salsolinol's enhancement of Gluergic transmission is dependent on TTX-sensitive Na^+^ channels.

### Salsolinol enhances evoked EPSCs (eEPSCs) in DA neurons

**Figure 4:**
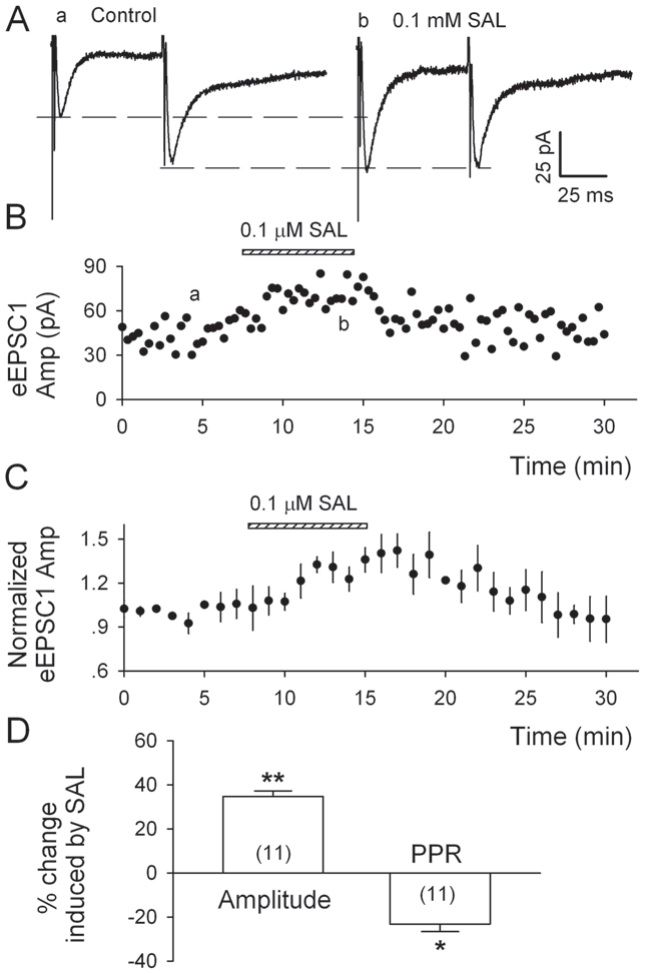
Salsolinol increases amplitude and decreases paired-pulse ratio (PPR) of evoked EPSCs (eEPSCs) recorded from p-VTA DA neurons. eEPSCs were recorded in the presence of bicuculline (10 µM) at a holding potential of −70 mV. A, Salsolinol (0.1 µM) sharply increased the peak amplitude of eEPSC_1_ and had minimal effect on that of eEPSC_2_ evoked by paired stimulation (at 50- ms interval) within the VTA, and hence reduced the PPR (EPSC_2_/EPSC_1_). B, Time course of salsolinol-induced enhancement of eEPSC_1_ amplitude in one experiment. C, Summary of salsolinol-induced changes (%) in eEPSC_1_ amplitude and PPR (Mean ± SEM, from eleven neurons). *P<0.05, **P<0.01, paired t-test for salsolinol vs. pre-salsolinol control.

The above data indicate that salsolinol increases Gluergic transmission via a presynaptic mechanism. To confirm this, we tested the effect of salsolinol on EPSCs evoked by pairs of stimuli (at 50 ms interval) applied by a local stimulating electrode in the presence of 10 µM bicuculline and at a Vh of –70 mV. These EPSCs were completely blocked by 20 µM DNQX (not shown), indicating that they were mediated by AMPA receptors. Salsolinol (0.1 µM) enhanced the amplitude of the first eEPSC (EPSC_1_, by 34.8±2.5%, n = 11; P<0.01, by paired t-test, Fig. 4A–C), but not the second eEPSC (EPSC_2_) and thus decreased the paired-pulse ratio (PPR = EPSC_2_/EPSC_1_, by 23.2±3.4% from 1.08±0.05 to 0.87±0.03; n = 11, P<0.05, paired t-test, Fig. 4A and C). It's well documented that changes in transmitter release generally affect the PPR [29], [30].

### Salsolinol-induced increase in EPSCs is abolished by a D_1_Rs antagonist, SKF 83566

**Figure 5:**
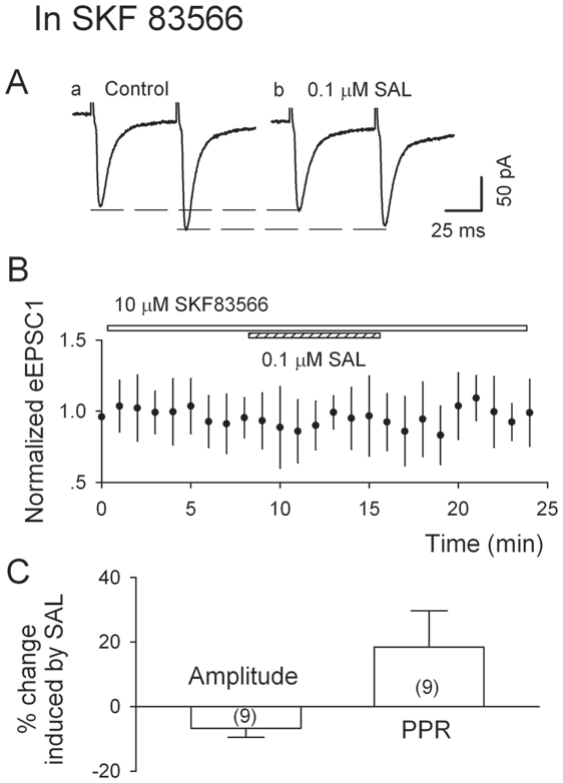
The D_1_R antagonist SKF 83566 eliminates salsolinol's augmentation of eEPSCs. A, Current traces show that salsolinol did not change the amplitude of eEPSC_1_ and PPR in the presence of 10 µM SKF 83566. B, Time course of salsolinol-induced changes of eEPSC_1_ amplitude in nine experiments. C, Summary of salsolinol-induced changes (%) in eEPSC_1_ amplitude and PPR in the presence of SKF 83566 (data from nine cells). *P<0.05, **P<0.01, paired t-test for salsolinol application vs. pre-salsolinol control.

In the VTA, D_1_Rs are expressed on Gluergic axons [31] but not on the soma of DA neurons [31], [32]. The activation of D_1_Rs increases glutamate levels in the VTA [20] as well as Gluergic transmission in the globus pallidus [33]. As was recently shown by our lab that ethanol-induced enhancement of Gluergic transmission onto VTA DA neurons involves D_1_Rs localized on the Gluergic terminals [21], [22], we wonder if salsolinol-induced enhancement of Gluergic transmission onto VTA DA neurons also involves D_1_Rs. We compared the effects of salsolinol on eEPSCs in the absence and presence of SKF83566 (10 µM). As depicted in Fig. 5A–C, in the presence of SKF83566, salsolinol (0.1 µM) failed to change either eEPSC_1_ amplitude (by −6.7±11.3%, n = 9, P = 0.6, paired t-test), or the PPR (by 18.4±2.8% of control, from 1.07±0.04 to 1.25±0.08; n = 9, P = 0.2, paired t-test). These results indicated that D_1_Rs are essential for salsolinol's enhancement of Gluergic transmission.

### Salsolinol-induced excitation of VTA DA neurons is attenuated by SKF 83566

**Figure 6:**
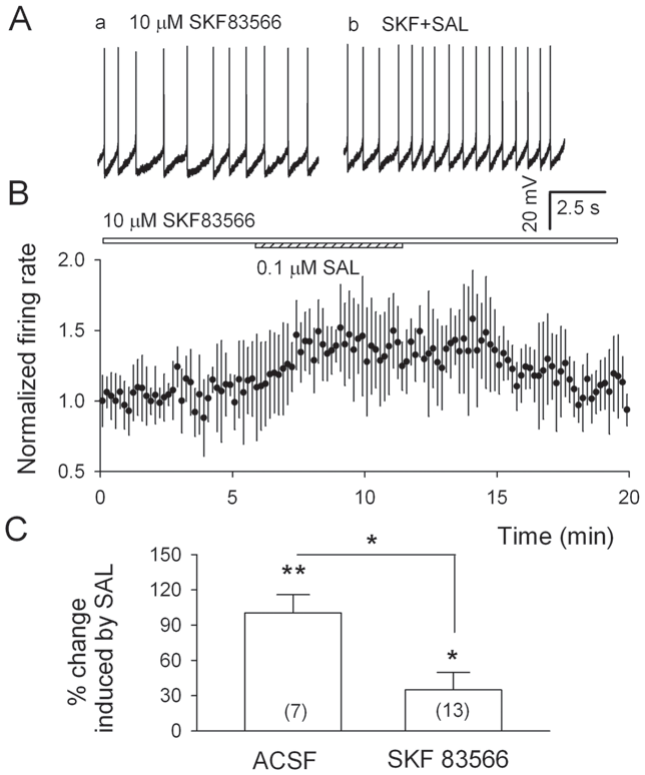
SKF83566 attenuates salsolinol-induced excitation of p-VTA DA neurons. A, Ongoing firing in the presence of SKF83566 (10 µM), recorded from a current-clamped p-VTA DA neuron, was increased by salsolinol (0.1 µM). B, Pooled data show that in the presence of SKF83566, salsolinol induced an increase in firing rate of p-VTA DA neurons from thirteen cells. C, Summary (means ± S.E.M.) of the effects of salsolinol (0.1 µM) on DA neuron firing rate in the absence (ACSF) and presence of SKF 83566 (SKF 63566). Numbers of cells are indicated in the brackets. *P<0.05 by un-paired t-test, salsolinol vs. SKF 83566+salsolinol.

To further assess the contribution of D_1_Rs to salsolinol's stimulation of DA neurons, we compared the effect of salsolinol on the ongoing discharges of p-VTA DA neurons in the absence and presence of SKF83566. In the presence of 10 µM SKF83566, salsolinol (0.1 µM) significantly increased the firing rate, but only by 34.9±14.8% (from 1.3±0.4 to 1.6±0.5 Hz, n = 13, P = 0.036, Fig. 6A–B), which is >50% smaller (Fig. 6C, P<0.05, by unpaired t test) than 100.3%, the increase induced by 0.1 µM salsolinol alone.

## Discussion

We show here the first electrophysiological evidence of salsolinol facilitation of Gluergic transmission onto p-VTA DA neurons in acute midbrain slices. Salsolinol (0.1 µM) robustly increased the spontaneous firing rate of DA neurons, which was substantially attenuated by APV and DNQX. Furthermore, salsolinol significantly increased the frequency of sEPSCs and the amplitude of the first eEPSCs, but reduced the paired-pulse ratio of eEPSCs. The effects of salsolinol on EPSCs were eliminated by the D_1_R antagonist SKF83566. Finally, SKF83566 attenuated salsolinol-induced increase in firing rate of p-VTA DA neurons. Taken together, these data indicate that salsolinol, via the activation of presynaptic D_1_Rs and consequently facilitation of Gluergic transmission contributes substantially to salsolinol-induced excitation of p-VTA DA neurons. Hence, salsolinol's effect on DA neurons involves activation of Gluergic transmission, in addition to the suppression of GABAergic transmission [10].

### Gluergic transmission is involved in Salsolinol-induced excitation of VTA DA neurons

Recently we have shown that salsolinol (0.01–1 µM) stimulated p-VTA DA neurons in brain slices of rats [10]. In this study, we showed that salsolinol's stimulation was substantially attenuated by APV and DNQX. This finding clearly points to the involvement of Gluergic transmission in salsolinol's action.

It is well accepted that VTA DA neurons are important for the response to reinforcement and reward signals. Accumulating evidence suggests that the input and output elements of DA neurons are essential components in the reward pathways. Gluergic transmission plays an important role in the effects of ethanol [34], [35]. In several brain regions, ethanol inhibits NMDA and non-NMDA glutamate receptors, as well as glutamate release [36]. However, there are also reports that ethanol increases glutamate release under some circumstances. Systemic administration of ethanol increases glutamate release in the nucleus accumbens of low-alcohol sensitive rats [37] and addiction-prone Lewis rats [38]. Studies on the central nucleus of the amygdale reveal that acute ethanol increases glutamate release only in rats receiving chronic ethanol treatment [39], [40]. We have previously demonstrated that ethanol enhanced Gluergic transmission to VTA DA neurons [21], [22]. In this study, we found salsolinol enhanced Gluergic transmission onto DA neurons. The application of APV+DNQX substantially attenuated salsolinol-induced increase in the firing rate of DA neurons, indicating that Gluergic activity contributes considerably to salsolinol-induced excitation of DA neurons. Moreover, TTX abolished the increase in the frequency of spontaneous EPSC induced by salsolinol, indicating that salsolinol's effect was dependent on TTX-sensitive Na^+^ channels. Salsolinol did not alter the amplitude of both sEPSCs and mEPSCs, indicating that salsolinol acted on the presynaptic site. The presynaptic loci were further supported by salsolinol's effects on evoked EPSCs: it enhanced the amplitude of the first EPSCs in response to the first stimuli of the paired stimuli but decreased their paired-pulse ratio.

### Role of D_1_Rs in salsolinol-induced excitation of VTA DA neurons

DA receptors consist of D_1_-like (D_1_ and D_5_ receptors) and D_2_-like (D_2_, D_3_ and D_4_ receptors) families. Both D_1_R and the D_2_R family (D_2_R in particular) [41] have been implicated in the mechanisms of drug dependence and abuse. Disruption of D_1_R gene expression [42] or administration of D_1_R antagonist [43] attenuates or prevents alcohol-seeking behavior. D_1_Rs are expressed on Gluergic axons [31] but not on the soma of VTA DA neurons [31], [32]. The activation of D_1_Rs increases glutamate levels in the VTA [20] as well as Gluergic transmission in the globus pallidus [33]. Previous works in our lab showed that ethanol may enhance glutamate release by potentiation of D_1_R function [21], [22]. In this study, we found that SKF83566 abolished salsolinol's enhancement of the eEPSCs, indicating that this enhancement is mediated by D_1_Rs. Salsolinol may increase the somatodendritic release of dopamine, which may retrogradely activate the D_1_Rs on the glutamate-releasing terminals, which in turn increases glutamate release and the excitability of DA neurons. Somatodendritic release of dopamine in the midbrain dopamine neurons is Na^+^ channel-dependent (see [44] for review). In keeping with this, we found that the increase in the frequency of spontaneous EPSCs was abolished by TTX. Interestingly, salsolinol is more potent than ethanol in enhancing Gluergic transmission as well as in excitation of DA neurons. The effective concentration of salsolinol in enhancing Gluergic transmission is much smaller than that of ethanol (peak effect: salsolinol: 0.1 µM vs ethanol: 40 mM [21], [22]. This is consistent with both in vivo and in vitro findings that DA system is highly sensitive to salsolinol [8], [9], [10]. We previously showed that ethanol-induced increase in the release of glutamate (EPSCs) in the VTA was eliminated when dopamine was depleted by the pretreatment of reserpine. Since salsolinol is the condensation product of dopamine and acetaldehyde, the metabolic product of ethanol, depletion of dopamine may prevent the formation of salsolinol. On the basis of the results of this study, we propose that the observation that ethanol failed to increase Gluergic transmission when dopamine was depleted may well be explained by the lack of salsolinol formation.

VTA DA neurons receive numerous inputs which in turn can modulate their eventual outputs. Integration of synaptic inputs and intrinsic properties sets the frequency and pattern of firing [45], [46], [47]. In this study, APV and DNQX, as well as SKF 83566, significantly attenuated but not totally abolished salsolinol-induced increase in the firing rate of VTA DA neurons. This is in line with our recent finding that salsolinol stimulates DA neurons partly through activation of μ opioid receptors, which leads to the inhibition of GABAergic transmission onto VTA DA neurons. It is worth noting that even in the presence of a mixture containing APV+DNQX+gabazine, salsolinol could still significantly increase the firing rate of p-VTA DA neurons (by 27.3±5.6%, n = 10, P = 0.03) (G Xie and JH Ye, unpublished observation). Furthermore, since the sum of the reductions induced by gabazine and by APV+DNQX in salsolinol-induced increase in firing rate of DA neurons is larger than one, the effects of salsolinol on GABAergic and Gluergic pathways may not be paralleled. This observation indicates the complex nature of salsolinol's effects. Other mechanisms underlying salsolinol-induced excitation of VTA DA neurons remain to be fully unveiled. On the bases of the findings of this study and our recent report [10], we propose that both Gluergic and GABAergic transmissions play essential roles in salsolinol-induced stimulation of VTA DA neurons. Our present work offers new insights into salsolinol's central mechanism under its rewarding properties.
